# A Retrospective, Observational Study of Catheter-Associated Urinary Tract Infection Events Post-Implementation of a Novel Urinary Catheter System with Active Drain Line Clearance and Automated Intra-Abdominal Pressure Monitoring

**DOI:** 10.3390/life12121950

**Published:** 2022-11-22

**Authors:** Patrick Brockway, David M. Hill, Vanessa Moll, Kelly Stanton, Manu L. N. G. Malbrain, Sai R. Velamuri

**Affiliations:** 1 Firefighters Burn Center, Regional One Health, Memphis, TN 38103, USA; 2 Department of Anesthesiology, Division of Critical Care Medicine, Emory University School of Medicine, Atlanta, GA 30322, USA; 3 Potrero Medical, Hayward, CA 94545, USA; 4 First Department of Anesthesiology and Intensive Therapy, Medical University Lublin, 20-954 Lublin, Poland; 5 Medical Data Management, Medaman, 2440 Geel, Belgium; 6 International Fluid Academy, 3360 Lovenjoel, Belgium

**Keywords:** catheter-associated infection, CAUTI, urinary tract infection, healthcare-associated infection, active drain line clearance, burns, intra-abdominal pressure, measurement

## Abstract

Objective: A quality improvement study to assess catheter-associated urinary tract infection (CAUTI) rate post-implementation of a bladder catheter with integrated active drain line urine clearance and automated intra-abdominal pressure monitoring in a burn intensive care unit (ICU). DESIGN: Eight-year retrospective before and after study (2015–2022). Setting: A single American Burn Association-verified Burn Center with 14 inpatient beds. Patients: Patients meeting criteria for admission to a Burn Center. Methods: Retrospective cohort study following the implementation of a novel urine output monitoring system with integrated drain line and urine clearance. Data from a 48-month (from January 2015–December 2018) historical control (period 1) were compared to data from a 28-month (from January 2020 to April 2022) post-implementation period (period 2). Pre- and post-implementation CAUTI event incidences were compared. Patients were transferred from outside hospitals with gravity bladder. A distinction in the chart between catheter types was impossible. Charts were reviewed to characterize patients with CAUTI events. Results: A total of 42 CAUTIs in 2243 patients were identified using the National Health and Safety Network (NHSN) definition during the analyzed period. There were 40 CAUTI events in period 1 and two CAUTIs in period 2. The incidence of CAUTI events pre-implementation was 0.030 (mean of 10 CAUTI events per year) compared to 0.002 (mean of 1 CAUTI event per year) post-implementation of an automatic drain line clearing UO monitoring system showing a significant reduction in CAUTI events (*p* < 0.01, risk ratio novel vs. gravity bladder catheter 0.071, 95% confidence interval: 0.017–0.294). Conclusions: CAUTIs were reduced in the period following the implementation of a novel urinary catheter system with an integrated active drain line and urine clearance in burn patients.

## 1. Introduction

Catheter-associated urinary tract infections (CAUTIs) are the most common hospital-onset healthcare-associated infections (HAI) in the United States. HAIs are essential quality and safety metrics publicly reported in the acute-care space and linked to hospital reimbursement by the Centers for Medicare and Medicaid Services (CMS) [1]. 

CAUTIs, represent approximately 9% of all HAIs, [2] and are associated with increased morbidity, mortality, and cost in intensive care units (ICUs) [3,4].

The National Healthcare Safety Network (NHSN) surveillance definition of CAUTI [5] includes the presence of an indwelling urinary catheter for at least two days, fever, and bacteriuria. CAUTI rates are reported to the Centers for Medicare and Medicaid Services (CMS). 

The most recent pooled mean CAUTI rate in ICUs in the United States was 2.5/1000 catheter days [6], with higher rates outside the US (pooled mean CAUTI rate of 4.8/1000 catheter days) [7]. CAUTIs are thought to impact patient outcomes and healthcare costs significantly. One study using administrative/claims data from 1990–2007 reported that CAUTI resulted in 2–4 days of excess hospitalization/episode, approximately 13,000 deaths, and excess healthcare costs of US $500 million annually in the US [8]. A more recent analysis (2013) concluded that CAUTIs accounted for only 0.3% of HAI costs in the United States, or approximately $28 million/year [9]. However, CAUTI data.

Burn patients are prone to intra-abdominal hypertension (IAH) and, depending on the total body surface area burned, receive frequent intra-abdominal pressure (IAP) measurements. IAP monitoring had most commonly been performed by instilling normal saline into the bladder and measuring a hydrostatic column [10,11]. Although some clinicians remain skeptical, this approach has mostly not been shown to promote CAUTI [12,13,14]. 

The objective of this study was to analyze CAUTI incidences before and after the introduction of a novel urinary catheter with an integrated active drain line clearance system for continuous urine output (CUO) and an automated intra-abdominal pressure (IAP) in a single-center burn intensive care unit.

## 2. Methods

### 2.1. Design and Patient Population 

This retrospective observational single-center study included all patients admitted to a single American Burn Association-verified Burn Center from January 2015 to April 2022. One Accuryn^®^ Monitoring System was implemented in early 2019, while six more Accuryn^®^ Monitoring Systems (Potrero Medical, Hayward, CA, USA) were implemented in October 2019.

Burn patients receive the Accuryn^®^ Monitoring System regularly, while overflow medical or surgical patients receive other gravity urinary catheter systems (Surestep™ Foley, BD). The lack of documentation in the electronic medical record (EMR) makes it impossible to determine which patients received the Accuryn^®^ Monitoring System versus other gravity urinary catheter systems. Furthermore, while charts of patients with CAUTI events were being reviewed, it was impossible to characterize the patients without CAUTIs. 

### 2.2. Catheter and Monitoring System

The Accuryn SmartFoley^®^ and Monitoring System (Potrero Medical, Hayward, CA, USA) are designed to reduce CAUTI by eliminating standing urine in the bladder and drainage system with an Active Drain Line Clearance and three one-way valves to eliminate urinary backflow. These features help to prevent retained urine (due to airlocks), reduce false oliguria, and enable real-time accurate continuous urinary output (CUO) [15]. 

Two different Accuryn SmartFoleys^®^ are used in the Burn Center: 1. CUO-only catheter and 2. CUO catheter that enables automatic intra-abdominal pressure (IAP) monitoring. Both Accuryn^®^ Systems are entirely closed systems where the actual urinary catheter comes connected to the tubing system, which is further secured through plastic wrapping. This closed system is interlocked into the monitoring system without any further intervention necessary on the drainage system itself. Bladder pressure as a surrogate of IAP is measured through a semi-flaccid balloon containing a pressure sensor at the tip of the urinary catheter [10,11]. In the Burn Center, IAP is measured if patients present with burns greater than 20 percent of total body surface area (TBSA), receive fluid resuscitation, suffer from 3rd-degree burns involving the anterior and/or posterior trunk, or have undergone escharotomies to the anterior trunk. IAP is then measured every 4 h [16,17,18]. 

### 2.3. Setting

The Burn ICU admits mainly burn patients but also serves as a medical or surgical overflow ICU. The Burn Center encompasses seven ICU beds, seven step-down beds, and three beds in the emergency department (ED). The entire Burn Center, including the ED, is a closed and locked unit for security and infection control reasons. Nurses are cross-trained to work in the burn ED, ICU, and Step-down. Step-down beds can become full ICU beds; in rare circumstances, ICU patients are housed in the step-down unit. The interdisciplinary team includes nurses, advanced practice providers, respiratory therapists, pharmacists, occupational therapists, physical therapists, dietitians, physicians, and student learners from multiple disciplines.

### 2.4. Ethical Considerations

The study was conducted in accordance with the study protocol, regulatory requirements, and good clinical practice. Following the Declaration of Helsinki, this minimal-risk study was approved by the University of Tennessee Health Science Center Institutional Review Board (IRB#22-08929-XP), including a waiver of written informed consent. 

### 2.5. Definition and Protocols

CAUTI events were defined by using the CDC NHSN definition [19]. CAUTI data are routinely collected and reported.

Per the Burn ICUs pathway, urine specimens were collected if the following applied:

1. Fever without evidence of another source;
2. Pain or burning while urinating;
3. Urgency;
4. Hematuria;
5. Costovertebral angle tenderness;
6. Burn Sepsis [20,21] without evidence of another source.

The urine sample is drawn immediately if a urinary catheter has been in place for less than 24 h. If the urinary catheter has been in place for more than 24 h, it is the Burn Center’s practice to replace the Foley catheter first and then draw a urine sample. Urine isolates were analyzed by the BD Phoenix^TM^ automated identification system (BD Diagnostics, Baltimore, MD), following the manufacturer’s recommendations.

IAP measurement protocols per the burn ICU

(a) IAP measurement set-up [10,11] using the gravity urinary catheter (protocol discarded when automatic IAP measurements with the Accuryn SmartFoley^®^ were introduced).
  Before performing IAP monitoring, the registered nurse (RN) is to ensure the following:
  1. The bedside monitor has been set up to perform IAP monitoring properly;
  2. The pressure bag attached to the tubing and transducer set-up for IAP monitoring is inflated to above 300 mm Hg;
  3. The tubing and transducer set-up has been properly primed with normal saline. Additionally, ensure that the tubing remains sterile by keeping the end covers intact;
  4. The following sterile supplies are at the bedside: 30 mL of sterile normal saline to instill into the bladder, a sterile 60 mL syringe, sterile gloves, sterile towels and amp, chlorhexidine, or alcohol preps for three separate cleaning steps. Nonsterile supplies needed at the bedside include clamps and amp, absorbent pads;
  5. The patient has an inserted Urinary Catheter with an access hub in place, and the catheter is draining urine appropriately.
(b) Performing IAP monitoring in the burn ICU (adopted and slightly modified from the WSACS recommendations [10,11]:
  1. Ensure the patient has been placed supine;
  2. Clean the access hub with chlorhexidine or alcohol. Rub the hub vigorously for at least 15 s (First clean);
  3. Clamp the Foley Catheter below the access hub;
  4. Don sterile gloves and establish a sterile field with sterile towels around the access hub using sterile technique;
  5. Prepare a sterile syringe with 30 mL of sterile normal saline;
  6. Clean access hub with chlorhexidine or alcohol using sterile technique. Rub the hub vigorously for at least 15 s. Allow the hub to dry for 30 s (Second clean);
  7. Instill 30 mL of sterile normal saline in the bladder;
  8. Clean access hub with chlorhexidine or alcohol using sterile technique. Rub the hub vigorously for at least 15 s. Allow the hub to dry for 30 s (Third clean);
  9. Attach monitoring tubing to access the hub. The system is now considered closed;
  10. Zero the IAP monitoring system on the bedside monitor;
  11. Obtain IAP;
  12. Unclamp Foley Catheter. Close off IAP monitoring tubing;
  13. Properly position the patient. Do not leave supine.
(c) Standard Operating Procedures (SOP) in the BICU concerning IAP monitoring:
  1. IAP monitoring is routinely performed every 4 h. Monitoring times can be increased or decreased based on the condition/or situation of the individual patient;
  2. It is the responsibility of the RN to promptly report IAPs of 20 mm Hg or greater to the provider;
  3. IAP monitoring tubing and normal saline used in the pressure bag are to be changed every 72 h;
  4. IAP monitoring tubing can be left attached to the Foley access hub but must be in the closed position when not in use;
  5. Sterile technique and proper cleaning are used when adding 30 mL of sterile saline for each IAP monitoring session.
(d) Automated IAP measurement: The patient is in the supine position, and active abdominal muscle contractions are absent. IAP is measured via button press. IAP monitoring is routinely performed every 4 h. Monitoring times can be increased or decreased based on the condition/or situation of the individual patient.

### 2.6. Statistical Analysis

Continuous variables are presented as medians with interquartile ranges following in brackets, [IQR], and categorical variables as percentages followed by the count in parentheses. Some patients had multiple distinct infections during their ICU stay, and these were maintained as separate CAUTI events. For the analysis, 2015 through 2018 are considered the pre-implementation phase and are termed Period 1, whereas 2020–2022 are considered the Accuryn^®^ Monitoring System post-implementation phase and are termed Period 2. 2019 data was considered the implementation period and excluded from the analysis. The Python programming language was used for all analyses with aid from the following packages: NumPy 1.19.1 [22], pandas 1.1.3 [23], matplotlib 3.3.1 [24], and scipy 1.5.2 [23]. The Fisher exact test was used to determine if there was an association between CAUTI incidence and the use of Accuryn^®^.

## 3. Results

The reporting period spanned 8 years (2019 data not analyzed) and included 42 distinct CAUTI events in 2243 patients. Table 1 depicts the demographics for patients with CAUTI events in each time period before and after implementation of the Accuryn^®^ Monitoring system.

The Burn Center took care of a mean of 359 patients per year. Three patient events were excluded as the prevalent organism detected was Candida albicans (candiduria is not included in the CAUTI event definition [19]). The most common organisms identified by urine culture were *Pseudomonas* spp. (45.2% (19) pre and 0.0% (0) post-implementation), *E. coli* (19.0% (8) pre and 50.0% (1) post-Accuryn^®^ implementation), as seen in Table 2.

**Figure 1:**
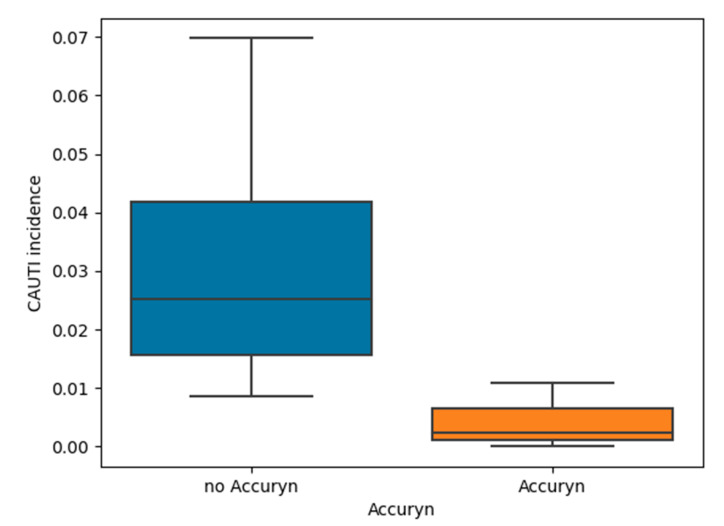
CAUTI incidence is lower in years following the implementation of the Accuryn^®^ Monitor relative to years prior; 2019 data was considered the implementation period and excluded from the analysis.

The incidence of CAUTI events in Period 1 was 0.030 (with a mean of 10 CAUTI events per year) compared to 0.002 (mean of 1 CAUTI event per year) in Period 2, showing a significant reduction in CAUTI events (*p* < 0.01, risk ratio Accuryn^®^ vs. gravity urinary catheter 0.071, 95% confidence interval: 0.017–0.294, shown in Figure 1). A boxplot of lower CAUTI incidence in years following the implementation of the Accuryn^®^ Monitoring System relative to years prior is shown in Figure 1. 

**Figure 2:**
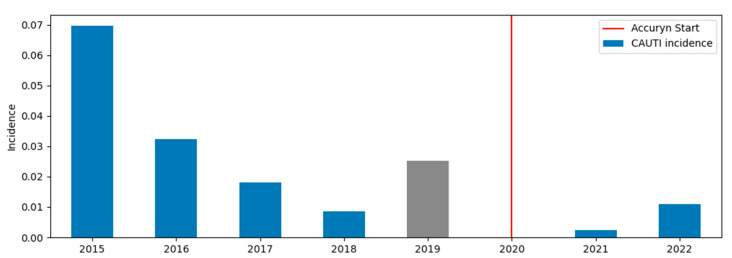
Time course of CAUTI events from 2015 to April 2022. 2019 (317 patients and 8 CAUTI events) was considered the implementation period and excluded from the analysis.

Median [IQR] urinary catheter dwell time to CAUTI events was 4.5 [2.0, 6.2] days pre- and 6.0 [5.5, 6.5] days post-novel catheter implementation. The time course of CAUTI events in the analyzed period is shown in Figure 2. 

## 4. Discussion

CAUTI is among the most common types of HAI and remains a major challenge for hospital safety and healthcare quality in ICUs [2,4,25]. A significant portion of these infections is preventable by using evidence-based strategies [25]. The current study aimed to determine if implementing a novel catheter system with an automated active drain line clearance reduced CAUTI events. The incidence of CAUTI events pre-implementation was 0.030 compared to 0.002 post-implementation, showing a significant reduction in CAUTI events. 

We acknowledge that CAUTI incidents seem to decline from 2015 through 2018, irrespective of the implementation of the Accuryn^®^ Monitoring System. However, in 2019 (not included in the analysis), CAUTI events increased again to 8 in 317 patients reversing the trend. We recommend future prospective trials to confirm the potential benefit of the Accuryn^®^ Monitoring System. 

Preventative measures for CAUTI events have mainly focused on limiting the use and early removal of indwelling urinary catheters. Other measures include the use of a condom or other external catheter or intermittent catheterization as alternatives to indwelling urinary catheters, proper aseptic technique during catheter insertion, adherence to optimal catheter maintenance throughout the duration of catheter use, and collaboration with nurses and other healthcare providers in the development and implementation of catheter removal protocols [25].

Especially in the critically ill burn patient population, which often requires accurate urine output monitoring and potentially IAP monitoring performed with bladder catheters, early removal of bladder catheters is not indicated. 

Bacteriuria is a risk factor for UTI; however, the frequency of progression from bacteriuria to CAUTI is low [26]. The development of CAUTIs and bacteriuria have been linked to bacterial biofilm formation on the inner surfaces of indwelling urinary catheters following its insertion [27,28]. Interestingly, antimicrobial coating of indwelling urinary catheters has not significantly reduced CAUTI rates [29]. The risk of developing a CAUTI is directly related to catheter dwell time [30]. The rate of development of catheter-associated bacteriuria is approximately 3 to 7% per day [25,31], and the likelihood of bacteriuria approaches 100% if a patient has an indwelling urinary catheter for ≥30 days [3]. Bacteriuria is a risk factor for UTI; however, the frequency of progression from bacteriuria to CAUTI is low [26].

It is frequently recommended to maintain the mechanical patency of the drain line and ensure that urinary drainage is unhindered [31,32]. In a study comparing urinary catheter systems with a single to a double valve to prevent urinary backflow, the time to colonization was 14 days and 21 days, respectively [33]. Catheterized urine (including standing urine in the bladder and drainage system) serves as a reservoir for multidrug-resistant organisms in the ICU [34]. Positive urine cultures are often treated with antibiotics. They are a critical driver of antimicrobial use [35], leading to increased patient harm from drug toxicity, C. difficile infection, and the risk of selecting even more resistant organisms. The avoidance of urinary backflow and standing urine in the bladder and drainage system, therefore, might delay bacterial biofilm generation and possibly delay and occurrence of a CAUTI event. With its Active Drain Line Clearance, the Accuryn SmarFoley^®^ and Monitoring System are designed to reduce CAUTI by eliminating standing urine in the bladder and drainage system. A recent small observational study described a reduction in CAUTI in a single ICU at a tertiary hospital with the Accuryn^®^ catheter and Monitoring System [36]. 

There has been some concern that the IAP monitoring technique of instilling normal saline into the urinary catheter and bladder might place patients at risk for CAUTI [13]. However, there are not a lot of data available to confirm this theory. In one small study, an open technique of measuring bladder pressure was found to be associated with a greater risk of CAUTI [14]. Two studies (adult and pediatric population) examining the risk of CAUTI in relation to bladder pressure measurements using a closed transducer technique showed no increased risk of CAUTI [13], and a closed system using the patient’s urine as a pressure transmitting medium also did not display an increased risk for CAUTI [12]. IAH will develop in most severely burned patients and may contribute to early mortality [37]. Mechanically ventilated burn patients display a relatively high incidence of IAH (64.7–78.6%) and ACS (4.1–28.6%) compared with other critically ill patients (34–49.8%) and undergo frequent IAP monitoring [16,38,39]. The use of continuous IAP monitoring will, in the future, likely detect higher rates of IAH and has many benefits beyond that are detailed elsewhere [40]. This is exemplified by the recent finding that IAH detected by continuous IAP monitoring in cardiac surgery patients was above 90% in the first 48 h after surgery [41]. 

In addition to optimizing patient care and outcomes by preventing CAUTIs, there are significant costs related to CAUTI events. In 2016, the reported attributable costs of CAUTIs were $876 (inpatient costs to the hospital for additional diagnostic tests and medications); $1764 (inpatient costs to Medicare for non-ICU patients); $7670 (inpatient and outpatient costs to Medicare); $8398 (inpatient costs to the hospital for pediatric patients); and $10,197 (inpatient costs to Medicare for ICU patients) [4]. Conservatively assuming costs of $1000–$5000 per CAUTI event, the implementation of the Accuryn^®^ Monitoring System in this single-center study resulted in cost savings of $9000–$45,000 annually.

## 5. Limitations

Our study has several limitations. First, it was performed in a single burn ICU in a large, academic tertiary care center, which may not be representative of other settings and limits external validity. Second, limitations inherent to retrospective observations using routinely recorded data include potentially limited control of unmeasured confounding and misclassification bias affecting the routinely recorded variables. Third, it was impossible to determine which patients received the Accuryn^®^ Monitoring System vs. gravity urinary catheter. Fourth, a lack of characteristic data for those patients that did not have CAUTI prevents adjusted analysis or looking at differences in patient characteristics. Lastly, the lack of total patient days for those patients not having a CAUTI prevented the rate analysis relative to patient days; CAUTI incidence was compared instead. 

## 6. Conclusions

In this small retrospective observational study, CAUTI events were significantly reduced after implementing a novel catheter and urine monitoring system in severely ill burn patients. Active drain line clearance, valves that prevent urinary backflow, and automated IAP measurements might be favorable features leading to a reduction in CAUTI events compared to gravity urinary catheters and allow not only CUO but also continuous IAP monitoring. However, further well-designed prospective studies are warranted to confirm the promising results of this study. 

## Figures and Tables

**Table 1:** Characteristics of patients with CAUTI events. Period 1 reflects the pre-implementation period, whereas period two is the post-implementation phase.

Characteristic	Category	Period 1, Median [Q25, Q75] or No. (%)	Period 2, Median [Q25, Q75] or No. (%)
Total patients (n)	2243	1317, 40 CAUTIs	926, 2 CAUTIs
Age (years)		49.0 [33.0, 61.5]	43.5 [34.2, 52.8]
BMI (kg/m^2^)		26.3 [23.4, 29.7]	24.1 [24.0, 24.2]
Gender	Male	52.5% (21)	100.0% (2)
	Female	47.5% (19)	0.0% (0)
Race	Caucasian	57.5% (23)	50.0% (1)
	African American	32.5% (13)	50.0% (1)
	Unknown	7.5% (3)	0.0% (0)
	Hispanic	2.5% (1)	0.0% (0)
Catheter day of CAUTI		4.5 [2.0, 6.2]	6.0 [5.5, 6.5]
Type of injury	Burn	85.0% (34)	100.0% (2)
TBSA%		24.2 [12.2, 70.0]	19.8 [18.4, 21.1]
	Smoke inhalation	5.0% (2)	0.0% (0)
	Electrical injury	2.5% (1)	0.0% (0)
	Frostbite	2.5% (1)	0.0% (0)
	Unknown	2.5% (1)	0.0% (0)
Comorbidities	Hypertension	13.4% (11)	16.7% (1)
	Smoker	11.0% (9)	0
	IDDM	8.5% (7)	0
	Anxiety	7.3% (6)	0
	Depression	6.1% (5)	0
	Cerebrovascular accident	4.9% (4)	0
	Hyperlipidemia	4.9% (4)	0
	Polysubstance abuse	3.7% (3)	16.7% (1)
	Hepatitis	3.7% (3)	0
	Coronary artery disease	3.7% (3)	0
	COPD	3.7% (3)	0
	Dementia	2.4% (2)	16.7% (1)
	Hypothyroidism	2.4% (2)	0
	Myocardial infarction	2.4% (2)	0
	Arthritis	2.4% (2)	0
	Seizures	2.4% (2)	0
	Asthma	2.4% (2)	0
	Post-traumatic stress disorder	1.2% (1)	0
	Alcohol abuse	1.2% (1)	16.7% (1)
	Degenerative disc disease	1.2% (1)	0
	Back pain	1.2% (1)	0
	GERD	1.2% (1)	0
	Anemia	1.2% (1)	0
	Congestive heart failure	1.2% (1)	0
	HIV	1.2% (1)	0
	Hyperthyroidism	1.2% (1)	0
	Autism	1.2% (1)	0
	Bipolar disorder	1.2% (1)	0
	Renal insufficiency	1.2% (1)	0
	Pancytopenia	0	16.7% (1)
	Cholelithiasis	0	16.7% (1)

IDDM, insulin-dependent diabetes mellitus; COPD, chronic obstructive pulmonary disease; GERD, gastroesophageal reflux disease; HIV, human immunodeficiency virus.

**Table 2:** Organisms found in urine cultures of patients with CAUTI events. Period 1 reflects the Accuryn^®^ pre-implementation period, whereas period two is the post-implementation phase.

Organism	Period 1, % (N)	Period 2, % (N)
Pseudomonas aeruginosa	45.2% (19)	0
Escherichia coli	19.0% (8)	50.0% (1)
VRE	4.8% (2)	0
Klebsiella pneumoniae	4.8% (2)	0
Enterococcus species	4.8% (2)	0
Achromobacter xylosoxidans	4.8% (2)	0
Acinetobacter Baumannii	4.8% (2)	0
Staphylococcus	2.4% (1)	0
Lactobacillus	2.4% (1)	0
Klebsiella oxytoca	2.4% (1)	0
Proteus vulgaris	2.4% (1)	0
Enterobacter cloacae	2.4% (1)	0
Enterococcus faecalis	0	50.0% (1)

VRE, vancomycin resistant enterococcus.

## Data Availability

The data presented in this study are available on request from the corresponding author. The data are not publicly available due to patient privacy.
